# Classification of Underwater Target Based on S-ResNet and Modified DCGAN Models

**DOI:** 10.3390/s22062293

**Published:** 2022-03-16

**Authors:** Zhe Jiang, Chen Zhao, Haiyan Wang

**Affiliations:** 1 School of Marine Science and Technology, Northwestern Polytechnical University, Xi’an 710072, China; ghgfhgfgh@mail.nwpu.edu.cn (C.Z.); hywang@sust.edu.cn (H.W.); 2 Key Laboratory of Ocean Acoustics and Sensing, Northwestern Polytechnical University, Ministry of Industry and Information Technology, Xi’an 710072, China; 3 School of Electronic Information and Artificial Intelligence, Shaanxi University of Science and Technology, Xi’an 710021, China

**Keywords:** underwater target classification, convolutional neural network, generative adversarial network

## Abstract

Underwater target classification has been an important topic driven by its general applications. Convolutional neural network (CNN) has been shown to exhibit excellent performance on classifications especially in the field of image processing. However, when applying CNN and related deep learning models to underwater target classifications, the problems, including small sample size of underwater target and low complexity requirement, impose a great challenge. In this paper, we have proposed the modified DCGAN model to augment data for targets with small sample size. The data generated from the proposed model help to improve classification performance under imbalanced category conditions. Furthermore, we have proposed the S-ResNet model to obtain good classification accuracy while significantly reducing complexity of the model, and achieve a good tradeoff between classification accuracy and model complexity. The effectiveness of proposed models is verified through measured data from sea trial and lake tests.

## 1. Introduction

Underwater target classification has been a hot topic driven by its important and general applications. However, many factors including complicated and variable marine environments, volatile modes of target related radiated noise, and lack of training samples have brought great challenges to the classifications of underwater targets. Radiated noise of an underwater target received by hydrophone is affected by navigational statuses, marine environment, etc. which leads to low classification accuracy.

Convolutional neural networks (CNNs) provide an efficient way to classify target in the field of image processing through local connection and weight planting sharing. CNN combines feature extraction and classifier design. Compared with traditional methods, CNN could avoid feature loss and dimension disaster and improve efficiency and accuracy of classification [1]. When applying CNN to underwater target classification, the normal way would be transforming target data into image data. Then, the image data are preprocessed and sent into CNN to classify target.

Ref. [1] proposed a deep competitive deep-belief network (CDBN) to learn underwater acoustic target features with more discriminative information from both labeled and unlabeled samples. By stacking the proposed competitive restricted Boltzmann machine, the network could adjust the activation level of the grouped hidden units by competitive learning. Ref. [2] presented an automatic target recognition approach for sonar onboard unmanned underwater vehicles (UUVs). Target features were extracted by a convolutional neural network (CNN) operating on sonar images, and then classified by a support vector machine (SVM) that was trained based on manually labeled data. Ref. [3] developed a new subband-based classification scheme to classify underwater mines and mine-like targets from the acoustic backscattered signals. The system consisted of a feature extractor using wavelet packets in conjunction with linear predictive coding (LPC), a feature selection scheme, and a backpropagation neural-network classifier. Ref. [4] used the idea of transfer learning to pre-train the neural network on the ImageNet dataset, and improved fish recognition performance correspondingly. Ref. [5] used the dataset of civil ships, and utilized the structure of CNN plus extreme learning machine (ELM) to classify underwater target. Ref. [5] utilized CNN to learn deep and robust features of underwater targets, followed by removing the fully connected layers. Then extreme learning machine (ELM) fed with the CNN features was used as classifier to conduct classification. Experiments on actual dataset of civil ships obtained recognition rate up to 93.04%. Ref. [6] used sparse autoencoder (SAE) to obtain spectral numbers from data of underwater targets, combining the softmax classifier. Ref. [7] proposed a classification and recognition method based on the time-domain second-order pooled CNN with the time–frequency joint attention mechanism.

In general, sample sizes of underwater targets are small, and different categories are imbalanced. This would cause serious problems when applying deep learning algorithm, which is not considered in the above investigations. To tackle the problem of small sample size, a deep learning model may be very complex and involve too many parameters with prohibitive computational burdens. Imbalanced categories may cause serious interference to the training of the model, and make the classification tend towards the class that dominates the data set.

In this paper, we have investigated underwater target classification based on a deep learning algorithm. To tackle the problems of small sample size and imbalanced categories of underwater target data, we have proposed the modified DCGAN model to augment the underwater target dataset by generating “fake” data with high quality and diversity based on real target data. We have proposed the S-ResNet model for underwater target classification by combining CNN with SqueezeNet, which is a popular type of lightweight neural network. We found that our proposed model obtains good classification accuracy while significantly reducing complexity of the model.

We summarize the contributions of this paper as follows:

1. We have proposed a modified DCGAN model to augment data for underwater targets, which could improve the quality and training stability for underwater targets with a small sample size.
2. We have proposed a S-ResNet model to obtain good classification accuracy while significantly reducing the complexity of the model.
3. Field experiments have been carried out with five different types of underwater targets, verifying the effectiveness of proposed models.

The structure of the paper is as follows. Section 2 introduces related works in the fields of data augmentation and classification models. Section 3 presents the materials and models of our proposed method, including the framework, the modified DCGAN model, and the S-ResNet classification model. Section 4 illustrates the performance our proposed models with experimental data. Finally, Section 5 draws conclusions and and discusses future work.

## 2. Related Works

### 2.1. Data Augmentation

In practical application, due to the uncertainty of underwater acoustic channels and sea conditions, as well as the diversity of underwater target types and working conditions, we may not be able to collect enough target data to train a neural network. There are roughly two ways to tackle the problem of insufficient data. The first is by modifying the loss function and class weight of the neural network, e.g., cost sensitive (Co Sen) function [8] and focalloss function [9], etc. Through dynamic change of weight and direction of gradient update, the ability of the network to learn useful features could be improved. The second is to augment the dataset. Common methods include affine transformation and noise addition. In [10], the authors proposed the Synthetic Minority Oversampling Technique (SMOTE), which expanded the small class samples through random sampling.

**Figure 1:**
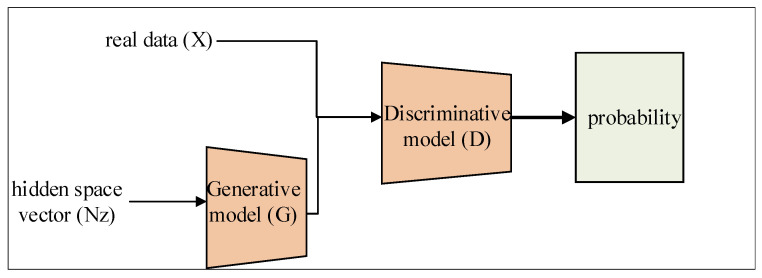
Construction of GAN.

Generative Adversarial Network (GAN), which has been applied to image processing, natural language processing, and speech recognition, is the most effective data augmentation tool. GAN can generate new data according to the distribution of real data and hence greatly improve the diversity and quality of the data [11]. GAN consists of a generative model (G), discriminative model (D), and loss function, as shown in Figure 1. The generative model would input the hidden space vector (Nz), and make it fit the distribution of real data (X) through continuous training and generating “fake” data similar to real data. The Discriminative model is essentially a dichotomy (0/1) and offers probability of input data. Ideally, weight parameters of generative and discriminative models are optimized by a dynamic change of loss function.

The application of GAN to underwater target classification mainly focuses on data augmentation for audio images, time spectrum images, and the visible light remote sensing images [12]. Ref. [13] proposed a conditional generated adversarial network (CGAN) for data augmentation, and used CNN for target classification. Ref. [14] used four GAN models to explore the effect of data augmentation: DCGAN, Auxiliary Classifier GAN (ACGAN), Least Squares Conditional GAN (LSCGAN), and Wasserstein Conditional GAN (WCGAN). The experimental results show that ACGAN is more suitable than other models in HRRP data augmentation. Ref. [15] presented a novel framework based on GAN to resolve the problem of insufficient samples of underwater acoustic signals. The audio samples were preprocessed to gray-scale spectrum images. Then, the data can fit the GAN and the complexity can also be reduced. An independent classification network outside the GAN was utilized to evaluate the generated samples by GAN. Ref. [16] trained DCGAN at the CIFAR10 dataset and tested the large-scale ImageNet dataset for the establishment of the proposed DCGAN. The generated and real image samples showed that the proposed DCGAN model works well with both datasets. The problems of GAN, however, are mode collapse and nonconvergence, which may significantly affect the performance of generated data. Therefore, many modified GAN models have been proposed to tackle this issue [17,18,19,20].

### 2.2. Classification Model

BackPropagation (BP) algorithm and sigmoid activation function were proposed by Geoffrey Hinton to effectively solve the nonlinear classification problem [21]. Then came the second wave of neural network. Later, it was discovered that sigmoid activation function has the problem of gradient vanishment. In 1998, Le Cun proposed the famous CNN model LeNet-5 [22]. In 2006, Geoffrey Hinton proposed a solution to the problem of gradient vanishment by combining unsupervised pre-training, initialization of weights and fine-tuning of supervised training [23]. In 2011, ReLU activation function was proposed, indicating the outbreak of deep learning [24]. In 2012, AlexNet was proposed and attracted the attention researchers [25]. In 2015, Szegedy proposed the inception module of parallel convolution, in which the form of convolution kernel was selected by the network [26]. A series of improvements for the inception module have been made subsequently [27,28]. The VGG took a different strategy by adopting a single convolution kernel with deep layer [29]. In order to further improve the network performance, researchers continue to deepen the depth of layers of CNN. However, the network showed a phenomenon of degradation. He K et al. proposed ResNet to solve the problem of degradation by way of a shortcut connection [30]. It turned out that the performance of ResNet is better with the same depth of network layers. A large number of deep neural networks have been successively proposed, including DenseNet [31], SENet [32], Res2Net [33], SqueezeNet [34], MobileNet [35], and ShuffleNet [36]. The diverse deep neural networks greatly expand the application of deep learning algorithms.

## 3. Proposed Underwater Target Classification Models

### 3.1. Framework

**Figure 2:**
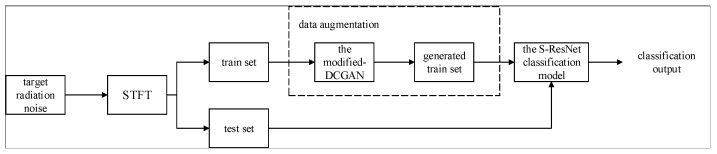
Block diagram of proposed underwater target classification method.

The block diagram of the proposed underwater target classification framework is shown in Figure 2. The collected radiated noises of a target are preprocessed by short-time Fourier transform (STFT), which can depict characteristics of different underwater targets in both time and frequency domains. The two-dimension results of STFT could be viewed as images, and different characteristics of STFT results corresponding to different underwater targets could be captured by different images. Thus, CNN-based classification models could be utilized to classify underwater targets.

We follow this methodology and divide the different target data in the form of time–frequency images randomly into train sets and test sets. The problem is, however, that given the price of obtaining data samples for different underwater targets, one may lack sufficient training data, especially for some types of targets. Considering we are dealing with time-*frequency images, naturally one could handle this problem by utilizing data augmentation such as GAN and DCGAN to generate new data to target. Unfortunately, we find that both GAN and DCGAN are not effective for underwater targets. In this paper, we have proposed the modified DCGAN model to augment data in training set for the target with limited samples. The augmented data are used to train and optimize the subsequent classification model. To deal with the classification of underwater targets more efficiently, we have also proposed S-ResNet classification model.

### 3.2. The Modified DCGAN

Although DCGAN has the powerful ability to generate new data with distribution similar to real data, the optimization of DCGAN may be disturbed by error characteristics in the process of learning and training, including mode collapse and checkerboard. GAN mode collapse is essentially a GAN training optimization problem. In this paper, we tackle the mode collapse problem by modifying the network architecture and optimizing the hyperparameter. Specifically, we have modified the architecture of DCGAN by tuning the last layer of convolution kernel in the generative model. When training the generative model, deconvolution is used for spatial sampling instead of a pooling layer. Time–frequency images of underwater targets are reconstructed using a set of convolution kernels and features. Through step-by-step deconvolution, the size of the images continuously expands in both length and width, whereas depth continuously decreases, until required size has achieved. Batch normalization (BN) is utilized to ensure unobstructed gradient flow, avoiding being affected by the initialization of weight parameters, and training performance could be improved. To avoid the problem of partly gradient saturation and to make the model more stable, the proposed generative model uses the ReLU activation function internally and the Tanh activation function in the data output layer. Furthermore, we find that the quality of generated data could be improved by optimizing the hyperparameter of adaptive moment estimation (Adam) optimization algorithm of generation and discriminant models. With respect to the optimization process for generative and discriminative models, we have selected the adaptive moment estimation (Adam) algorithm to improve stability. Specifically, we can prevent shock and instability by changing the parameter from 0.9 to 0.5 in the Adam optimization algorithm.

**Figure 3:**
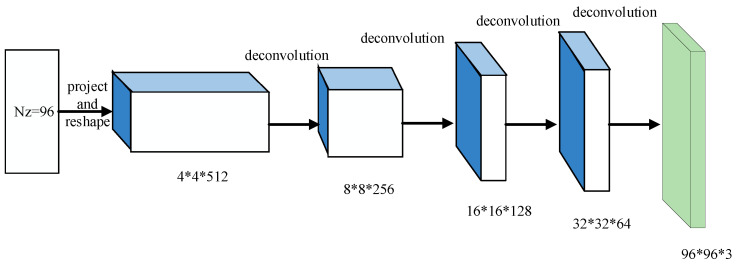
Construction of generative model.

In our modified-DCGAN, the values of convolution kernel size and stride during deconvolution operation are optimized to reduce the checkerboard effect. Through a large number of training and optimization, the size of the convolution kernel and stride are set as 4×4 and 2 for deconvolution in the generative model, respectively, whereas corresponding values are set as 5×5 and 3 in the last layer of the generative model to reduce the checkerboard effect. The generative model of proposed modified-DCGAN is shown in Figure 3, and the specific structure is illustrated in Table 1.

**Figure 4:**
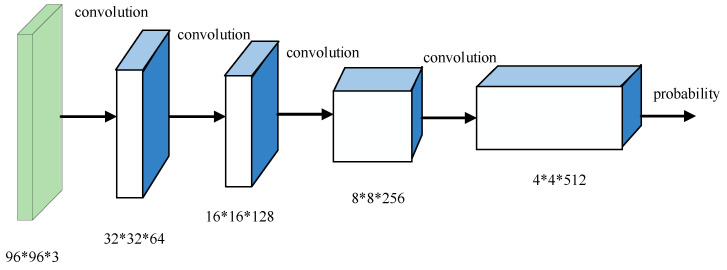
Construction of discriminative model.

In our discriminative model, a convolutional layer with the stride greater than 1 is used to replace the pooling layer for spatial subsampling. The last layer is flattened and sent to the output layer to preserve position information as much as possible. Similar to the the generative model, the discriminative model uses the Leaky ReLU activation function internally to maximize retention of information from the previous layer and update the negative ladder information. Sigmoid activation function is used only in the data output layer. BN is utilized to stabilize the learning process. The discriminative model used in this paper is shown in Figure 4, and Table 2 shows the specific structure of proposed discriminative model.

### 3.3. The Classification Model

In this paper, we have proposed the S-ResNet model for underwater target classification by combining a CNN model with a lightweight neural network. The proposed model is expected to effectively reduce the number of network parameters and computational complexities without deteriorating the performance of underwater target classification.

**Figure 5:**
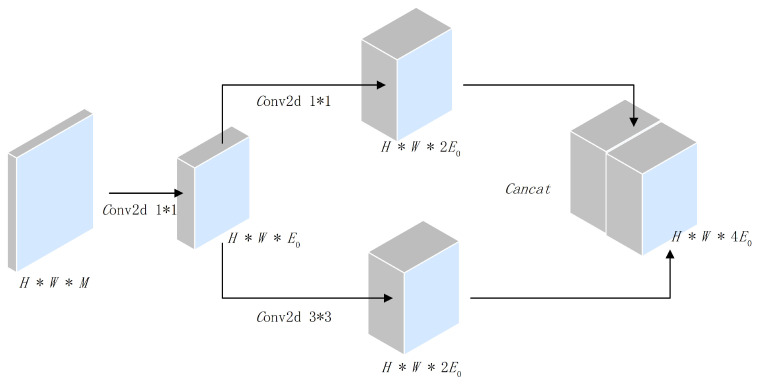
The construction of S-ResNet classification model.

**Figure 6:**
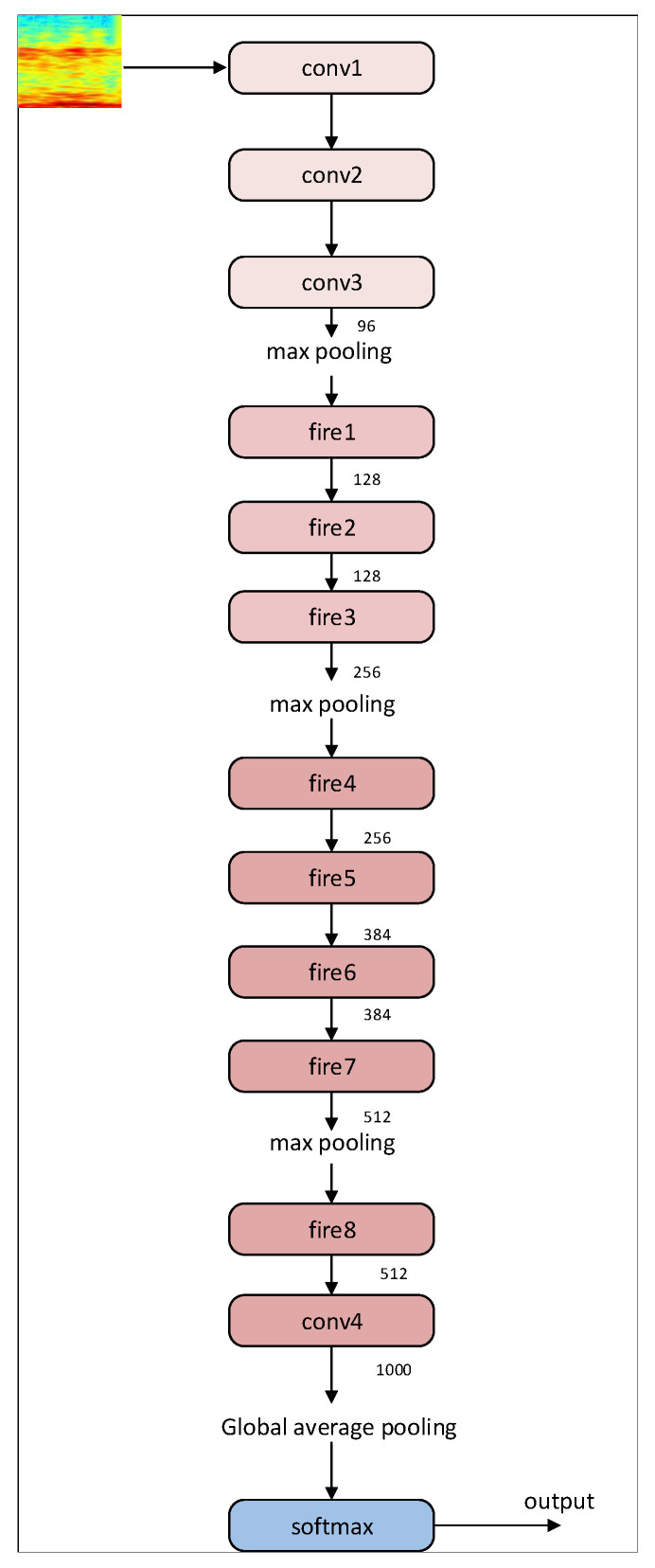
The fire module of S-ResNet classification model.

The specific structure of proposed S-ResNet classification model is shown in Figure 5 and detailed as follows. The 7×7 convolution layer in the first layer of the SqueezeNet network model is decomposed into 3×3 convolution layers, by which the number of model parameters could be significantly reduced without sacrificing classification performance. Inspired by the idea of fire module in the SqueezeNet, we have designed a new fire module as the constructive unit block for the S-ResNet classification model, as shown in Figure 6. In the designed fire module, the input size is H×W×M, in which *H*, *W*, and *M* represent the length, width, and number of channels of the input sample data, respectively, and the output characteristic graph is $H\times W\times (4E_{0})$, in which $E_{0}$ denotes the number of convolution kernels (the number of convolution kernels with 1×1 and 3×3 are both $2E_{0}$). Note that, in our S-ResNet model, we have $E_{0}=16$. The compression ratio parameter in the fire module in this section is set as 0.25.

Note that the number of convolution kernels of the proposed S-ResNet model is reduced compared with SqueezeNet, thus the computational complexity is also reduced. Furthermore, the ratio of squeeze layer to expand layer of the S-ResNet model is 1:4, while in the original SqueezeNet it is 1:8. By increasing the ratio, we can obtain a better tradeoff between classification performance and computational complexity. The ratio of the number of 3×3 convolution kernels in the expanded layer to the total number of convolution kernels is a hyper parameter, which is set as 0.25 in our model, showing the tradeoff between the performance and complexity of the model.

The S-ResNet classification model can further improve the performance of quantitative neural network without increasing the number of parameters of the CNN through convolutional kernel decomposition and compression ratio hyperparameter. Compared with the classical convolutional neural network, the S-ResNet classification model has the advantages of fewer parameters.

The cross entropy loss function is used in conjunction with softmax in our model. The specific structure of S-ResNet classification model is shown in Table 3.

## 4. Experimental Results

**Figure 7:**
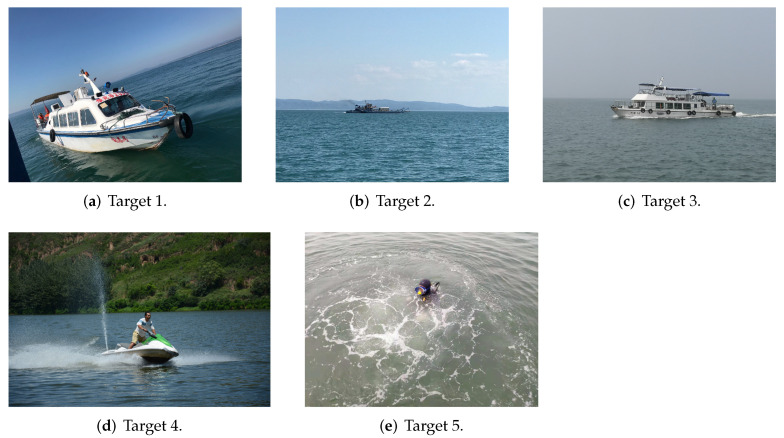
Five types of different targets.

The experiments were conducted at three different locations in China, namely the Danjiangkou Reservoir in Henan Province, the Yangjiahe Reservoir in Shaanxi Province, and Jiao Zhou Bay in Shandong Province. The collected data correspond to five different types of targets, namely a speedboat, two different types of ferries, a motorboat, and a frogman, as shown in Figure 7.

Collected data of each type of target are processed with short-time Fourier transform (STFT). The resulted time–frequency diagrams are taken as dataset *S*, which is randomly divided into train set $S_{1}$ and test set $S_{2}$ in accordance with preset ratio of 7:3. The original resolution of the generated time–frequency diagram is 1495×895, and is reduced to 224×224. This is the standard resolution of classic CNN-based models, and the computational complexity can be reduced with lower resolution.

**Figure 8:**
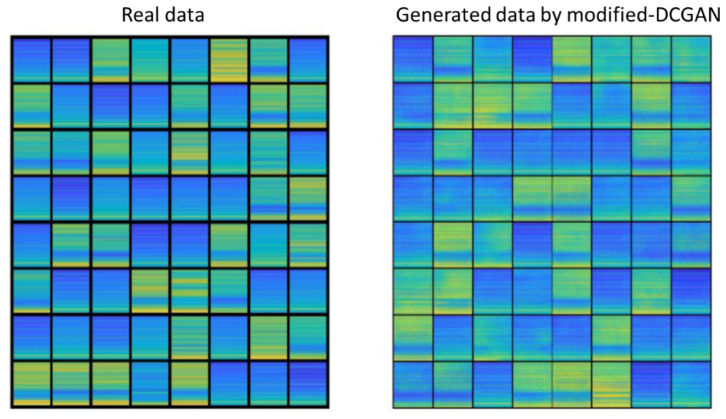
Generated data through proposed modified DCGAN.

**Figure 9:**
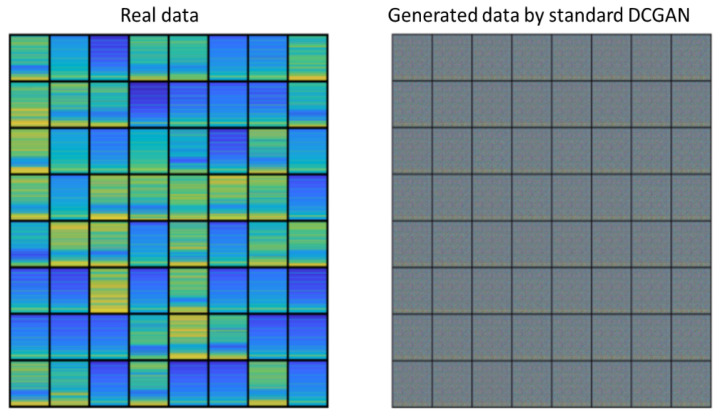
Generated data through standard DCGAN.

As the collected data from the frogman are very limited, we test our proposed modified DCGAN and standard DCGAN models on the frogman dataset. Parameter settings of both models are shown in Table 4. The graphs from real data and generated graphs through proposed modified DCGAN with the resolution 96×96 are shown in Figure 8, whereas the generated graphs by standard DCGAN with the resolution 64×64 are shown in Figure 9. We put the 64 generated graphs together for comparison.

As Figure 9 shows, the standard DCGAN model fails to generate effective data, and all generated data are same, indicating mode collapse and checkerboard artifact. These problems may be caused by insufficient feature learning of the standard DCGAN model when generating data. On the other hand, it can be seen from Figure 8 that positions of spectral line are approximately correct. Furthermore, the generated graphs from proposed modified DCGAN model and real data look very similar, indicating high quality and good diversity of the generated data.

Next, we quantitatively evaluate our model through two commonly used indicators, namely Frechet inception distance (FID) and inception score (IS), in Table 5. Essentially, FID measures the difference between the real data and generated data, while IS indicates the quality and diversity of generated data. As shown in Table 5, a smaller FID value indicates the data generated through our modified DCGAN model are closer to the real data, whereas a larger IS value suggests better quality and diversity.

**Figure 10:**
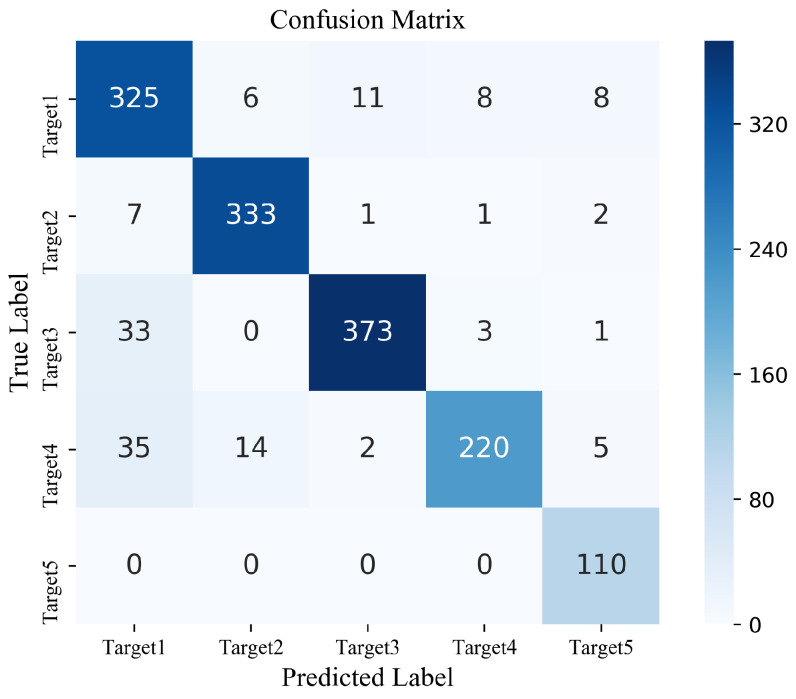
The confusion matrix with data augmentation.

We augmented data corresponding to the frogman by 200 extra samples with the proposed modified DCGAN model. The numbers of data samples with augmentation for different targets are shown in Table 6. The original train set S1 is supplemented by generated data, and proposed S-ResNet classification model is tested on test set. Parameter settings of proposed S-ResNet classification model are shown in Table 7. The obtained confusion matrix is shown in Figure 10, and the classification performance is shown in Table 8.

For comparison, we provide the classification performance without data augmentation in Table 9. Clearly, classification performance is improved by utilizing a modified DCGAN model, indicating the effectiveness of the proposed model. Note that, although we only augment the dataset corresponding to the frogman, the whole classification performance could be improved.

**Figure 11:**
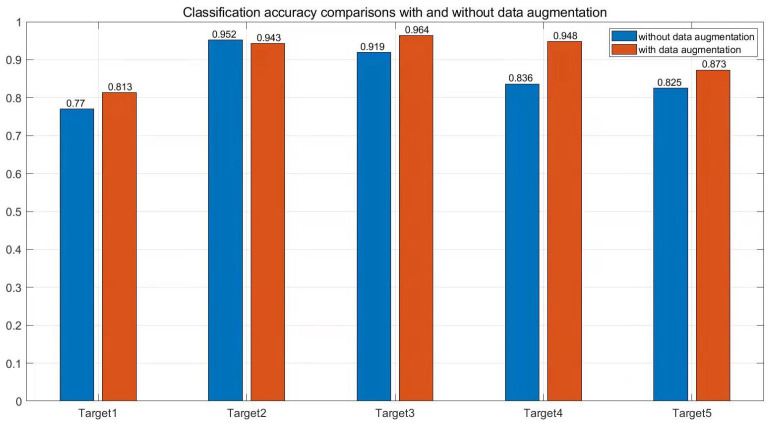
Classification accuracy comparisons with and without data augmentation.

The effect of data augmentation for each type can be observed more clearly in Figure 11, where we exhibit the classification accuracy comparisons of five different targets without and with data augmentation through the modified-DCGAN model. Naturally, the classification accuracy of the frogman is significantly improved from 83.6% to 94.8% with more generated trained data. Furthermore, the classification accuracies of other targets are increased by 4.3–4.8% or maintained, although no extra trained data are generated for these targets.

Then, we compare our S-ResNet model with other classification algorithms. Specifically, we test four classical machine learning classification algorithms, decision tree, KNN, random forest, and multi-classification SVM, using the datasets of five types of underwater targets obtained from the experiments. Classification comparisons of different classification algorithms are shown in Table 10.

As shown in Table 10, our S-ResNet model outperforms other algorithms in terms of classification accuracy of each target. The overall classification accuracy is increased by 6.9–10.5%.

Furthermore, we have tested the up-to-date CNN-based models, including ResNet-18, ResNet-34, ResNet-50, VGG-16, DenseNet, AlexNet, and SqueezeNet, based on the datasets of five types of underwater targets obtained from the experiments. We have compared our model with these models in terms of accuracy, number of parameters, FLOPs, and Epoch. The comparisons of classification performance with different models are shown shown in Table 11.

Clearly, the proposed model improves classification accuracy compared with SqueezeNet, which is a lightweight CNN classification model, while maintaining the low complexity of the model. On the other hand, the proposed model significantly reduces complexity compared with other classification models, indicated by the number of parameters, FLOPs, and epoch, while keeping excellent classification accuracy. These results exhibit that the proposed model achieves a good tradeoff between classification accuracy and complexity.

## 5. Conclusions and Future Work

Underwater target classification has general and important applications in both military and civil fields. Traditional manual-feature-based classification methods do not work well due to complicated and variable marine environments. Given their excellent performance in the field of image processing, CNN and related deep learning algorithms are expected to contribute to underwater target classifications. However, problems including small sample size of underwater targets and low complexity requirement need to be carefully handled for practical applications of deep learning algorithms. This paper proposes the modified DCGAN model to augment data for targets with small sample sizes, which could enhance classification performance under imbalanced category condition. The S-ResNet model has also been proposed to achieve a good tradeoff between classification accuracy and model complexity, as demonstrated with the data of five different types of underwater targets collected from sea trials and lake tests. Although we mainly focus on data augmentation of the target with the least samples, which is the frogman in our experiment, the proposed model could in principle be used to augment data of any target. It is interesting to further test our model for other targets. Furthermore, although our model has significantly reduced model complexity, the complexity needs to be further reduced for practical applications, due to the very limited energy resources and inconvenience of changing batteries in an underwater environment. We will focus on these problems in future investigations.

## Figures and Tables

**Table 1:** Specific structure of the generative model.

	Kernel	Stride	Padding	BN	Activation Function	Output
Input	4×4	1	0	Y	ReLU	4×4
Conv1	4×4	2	1	Y	ReLU	8×8
Conv2	4×4	2	1	Y	ReLU	16×16
Conv3	4×4	2	1	Y	ReLU	32×32
Conv4	5×5	3	1	Y	Tanh	96×96

**Table 2:** Specific structure of the discriminative model.

	Kernel	Stride	Padding	BN	Activation Function	Output
Input	5×5	3	1	Y	Leaky-ReLU	32×32
Conv1	4×4	2	1	Y	Leaky-ReLU	16×16
Conv2	4×4	2	1	Y	Leaky-ReLU	8×8
Conv3	4×4	2	1	Y	Leaky-ReLU	4×4
Conv4	4×4	1	0	Y	Sigmoid	

**Table 3:** Specific structure of the S-ResNet classification model.

	Input-Size	Kernel-Size	Depth	$S_{1\times 1}$	$E_{1\times 1}$	$S_{3\times 3}$
Input	224×224×3	3×3/2×96	1			
Conv1	111×111×96	3×3/1×96	1			
Conv2	111×111×96	3×3/1×96	1			
Conv3	111×111×96	3×3/1×96	1			
Maxpool	111×111×96	3×3/2	0			
fire1	55×55×128		2	32	64	64
fire2	55×55×128		2	32	64	64
fire3	55×55×256		2	48	96	96
Maxpool4	27×27×256	3×3/2	0			
fire4	27×27×256		2	48	96	96
fire5	27×27×384		2	64	128	128
fire6	27×27×384		2	64	128	128
fire7	27×27×512		2	128	256	256
Maxpool8	13×13×512	3×3/2	0			
fire8	13×13×512		2	128	256	256
Conv4	13×13×1000	1×1/1×1000	1			
GAP	1×1×5	13×13×1	0			

**Table 4:** Hyperparameter settings of modified DCGAN model.

Hyperparameter	Value
Input-dimension	96
Batch-size	64
Epoch	100
Adam	0.5, 0.999
Learning-rate	0.0002
Leaky ReLU	0.2

**Table 5:** Comparison between modified and standard DCGAN on IS and FID Indicators.

Model	FID	IS
Modified DCGAN	260.6227	1.162±0.034
Standard DCGAN	330.8796	1.000±0000

**Table 6:** Number of samples in dataset with data augmentation.

	Train Set	Test Set	Total
Target 1	929	400	1329
Target 2	832	353	1185
Target 3	890	387	1277
Target 4	548	232	780
Target 5	434	126	560

**Table 7:** Hyperparameter settings of S-ResNet classification model.

Hyperparameter	Value
Batch-size	32
Epoch	30
SGDM	0.999
Learning-rate	0.001
Leaky ReLU	0.2

**Table 8:** The classification performance with data augmentation.

	Target 1	Target 2	Target 3	Target 4	Target 5
Test Accuracy	0.92				
Precision	0.813	0.943	0.952	0.948	0.902
Recall	0.908	0.968	0.903	0.800	1
F1-score	0.858	0.955	0.927	0.868	0.948

**Table 9:** The classification performance without data augmentation.

	Target 1	Target 2	Target 3	Target 4	Target 5
Test Accuracy	0.86				
Precision	0.770	0.952	0.920	0.836	0.825
Recall	0.800	0.928	0.906	0.773	0.981
F1-score	0.785	0.940	0.913	0.803	0.896

**Table 10:** Accuracy comparisons of S-ResNet with different algorithms.

	S-ResNet	Decision Tree	KNN	Random Forest	Multiclassification SVM
Target 1	0.813	0.674	0.795	0.698	0.752
Target 2	0.943	0.744	0.769	0.825	0.689
Target 3	0.964	0.750	0.821	0.874	0.782
Target 4	0.948	0.653	0.691	0.701	0.710
Target 5	0.873	0.692	0.658	0.684	0.672
Overall	0.92	0.815	0.831	0.851	0.822

**Table 11:** Comparisons of classification performance with different models.

	Accuracy	Parameter (M)	Flops (G)	Epoch (s)
S-ResNet	0.92	1.03	0.8	50
SqueezeNet	0.87	1.25	0.82	44
ResNet-18	0.93	11.7	1.82	46.2
ResNet-34	0.93	21.8	3.67	64.7
ResNet-50	0.93	25.63	3.87	72
VGG-16	0.94	138.37	15.48	73.8
DenseNet	0.90	8.06	2.85	70
AlexNet	0.90	61.1	0.71	42.3

## Data Availability

Not applicable.
